# Nutritional Intake and Meal Composition of Patients Consuming Texture Modified Diets and Thickened Fluids: A Systematic Review and Meta-Analysis

**DOI:** 10.3390/healthcare8040579

**Published:** 2020-12-21

**Authors:** Xiaojing Sharon Wu, Anna Miles, Andrea Braakhuis

**Affiliations:** 1 Faculty of Medical and Health Sciences, Discipline of Nutrition, The University of Auckland, Auckland 1010, New Zealand; a.braakhuis@auckland.ac.nz; 2 Faculty of Science, School of Psychology, Speech Science, The University of Auckland, Auckland 1010, New Zealand; a.miles@auckland.ac.nz

**Keywords:** older adults, texture-modified diet, dysphagia, deglutition, deglutition disorders, nutrition, foodservice

## Abstract

Texture-modified diets (TMDs) play an important role in ensuring safety for those with dysphagia but come with risks to nutrition and quality of life. The use of TMDs has been addressed with the increasing prevalence of dysphagia in previous decades. However, there is limited literature that investigates the nutrition perspectives of TMD consumers. This review summarises the nutrition outcomes of adults consuming TMDs and thickened fluids (TFs) and identifies the limitations of TMD and TF productions. A systematic database search following PICO criteria was conducted using Cochrane Central (via Ovid), MEDLINE, CINAHL, EMBASE, and Scopus databases. Nutrition intake, meal consumption, adequacy, and meal composition were identified as relevant outcomes. 35 studies were included for analysis. Consumption of TMDs demonstrated a poorer intake compared to regular diets, in particular significant in energy and calcium. Meta-analysis of mean differences showed favourable effects of shaped TMDs on both energy (−273.8 kJ/d; 95%CI: −419.1 to −128.6, *p* = 0.0002) and protein (−12.4 g/d; 95%CI: −17.9 to −6.8, *p* < 0.0001) intake compared to traditional cook-fresh TMDs. Nutrition intake was compromised in TMD consumers. Optimisation of nutrition intake was achievable through enrichment and adjusting meal texture and consistency. However, the heterogeneity of studies and the missing verification of the consistencies lead to difficulty in drawing conclusions regarding particular texture or intervention.

## 1. Introduction

Dysphagia is defined as a difficulty in swallowing, characterised by impaired movement or obstruction, anywhere from the mouth to the stomach [1]. Dysphagia is more prevalent in older adults [2,3] and is a secondary condition associated with dementia, stroke, Parkinson’s disease, head and neck cancers, and many other neurological conditions [1,4,5]. Negative complications may be acute in onset, such as aspiration pneumonia caused by aspiration, or sudden death caused by choking [6]. Older patients with dysphagia are at high risk of chronic complications that influence patients’ health, nutritional status, frailty, mental status and quality of life [6,7,8]. 

Dietary modification by adapting the texture, consistency, and viscosity of food and drinks guided by speech-language therapists [4,9] is a common clinical approach for patients with dysphagia [6,10]. The use of texture-modified foods (TMDs) can reduce aspiration and choking risks in patients with oropharyngeal dysphagia. TMDs ease chewing and swallowing efforts for older adults with degenerated masticatory ability and dental loss [11]. TMDs are commonly prepared by chopping food into smaller pieces, tenderising the food, or adding liquid to blend the food into a smoother texture. Thickened fluids (TFs) are modified liquids using thickening agents. This higher viscosity slows the liquid flow reducing the risk of aspiration in some patients [12]. Inadequate nutritional intake has been well reported in patients with dysphagia in the last few decades [13,14,15]. Older adults prescribed TMD are at a higher risk of inadequate oral intake and require increased time and effort to swallow in comparison to older adults on regular diets. Painful swallowing and unappealing food aesthetics are contributing factors to poor oral intake, leading to malnutrition and dehydration [15,16,17]. 

Increasing demands for TMDs are found in the aging population as evidenced by the high prevalence of older adults with dysphagia in hospitals, aged-care, and community, with current rates ranging from 30% to 60% [8]. Studies indicate the frequent use of TMDs as the predominant intervention for older patients with dysphagia in both hospital and aged-care [18,19]. Despite the increase in TMDs, there has been a paucity of studies investigating the effect texture modification has on oral intake and nutrition density. It is important to investigate food texture to maximise eating experience and nutrition intake. Underpinning the value of providing TMDs, is the ability to standardise these diets from a global perspective. Studies investigating TMDs are limited partly due to the absence of global standardised terminology and measurement prior to the introduction of the International Dysphagia Diet Standardisation Initiative (IDDSI) in 2015 [3,20].

There have been multiple attempts to optimise nutrition intake in aged care, primarily by providing nutritionally dense food to people on TMDs in the form of supplementation or fortification [21]. Other oral nutrition interventions include shaping and moulding the food closer to regular food by adding thickening agent or enrichment powder (such as fortification); adjusting the texture and consistency complied to an individual’s diagnosed level of TMD; and offering nutritious in-between-meals [22]. Previous reviews have focused on swallowing safety and secondary complications of dysphagia [12,23,24]. There has been limited research summarising the existing evidence of nutrition outcomes on TMDs and TFs. Understanding the nutritional adaption in older patients with dysphagia and current foodservice practice will support health outcomes and research moving forward. This systematic review and meta-analysis aimed to evaluate the nutrition intake and adequacy of older adults consuming TMDs and TFs, and identify the gaps in meal production.

## 2. Materials and Methods

This study was conducted following the PRISMA-P reporting checklist. The protocol was registered on PROSPERO CRD42019134897.

### 2.1. Selection Criteria

Studies were required to include TMDs and/or TFs as an intervention. Study designs included randomised control trials (RCTs), cohort, pre-post, cross-sectional observational, and experimental studies. Case studies, reviews, expert opinions, conference paper, and uncompleted clinical trials were excluded. Only studies published in English were included. Study eligibility was defined using PICO framework. Eligible study participants were 18 years or older consuming TMDs or TFs in any type of setting, including community, hospital, and aged-care or foodservice producing TMDs for older adults. A clear fluid diet was excluded for the reason that it is commonly prescribed to patients with gastrointestinal disease to minimize digestion rather than an intervention diet for the dysphagic population [25]. The selected studies assessed any of the following nutritional parameters: macronutrient and micronutrient intake, meal consumption, nutrition adequacy assessed by actual intake comparing to national dietary reference values (DRVs) for each available nutrient and meal content (nutrient compositions or texture/consistency).

### 2.2. Data Sources

The authors searched electronic databases for eligible studies published before July 2019, including Cochrane Central (via Ovid), MEDLINE, EMBASE, SCOPUS, and CINAHL. Searching key terms for food and fluid was used in combination with terms related to texture modified (Supplementary data, Table S1). Search results from all databases were then transferred into one electronic library (Mendeley Desktop 1.19.4) [26]. One author merged and deduplicated the records. The reference lists from the full-text articles retrieved were then reviewed to identify additional relevant studies. The same screening process of eligibility was conducted.

### 2.3. Data Collection and Analysis

One author conducted initial titles and abstracts screening using the inclusion and exclusion criteria. Full-texts were retrieved for the eligible studies and reviewed by two authors for final inclusion. One author conducted the data extraction using a structured abstraction tool (specifically designed data extraction form developed in Microsoft Excel for Office 365, version 1902 [27]), recording study design, sample size, age, participant conditions, settings, year, country of origin, type of TMD or thickened fluid, study method, assessment tool, outcome measurement, findings, and limitations. When the data was not sufficiently presented in the manuscript, authors were contacted for clarification and full data sets were requested. Baseline data were used in cohort studies when no follow-up measurement was available. 

For a focus on nutritional outcomes, eligible studies were grouped according to outcome measurement (nutrition intake, meal consumption, nutrition adequacy, nutrient composition, and texture of the meal) for analysis. Subgroup analysis comparing TMDs against regular diet and different interventions were listed under each outcome. Sample size, mean value, and 95% confidence interval were extracted for both control and intervention groups. Variation in study outcomes due to inconsistency between studies was described by the Higgins score (I^2^), with 75% considered as high heterogeneity across studies [28]. Random and fixed effects model was chosen for high (I^2^ ≥ 75%) and low to medium (I^2^ < 75%) heterogeneity respectively [29]. Study intervention, data collection methods, participant characteristics, and settings were all considered potential contributors to the variations between studies. A funnel plot was used to evaluate publication bias. Research suggested meta-analysis should not combine different study designs to avoid misleading results [30]. Meta-analysis was conducted using Review Manager 5.3 [Cochrane, London, UK] where three or more studies used the same study design (either RCTs or observational studies) with the same intervention and outcome measurements [31]. Pooled data are presented as mean difference, 95% confidence interval, and *p*-value.

Study qualities were graded using Academy of Nutrition and Dietetics Quality Criteria Checklist (QCC) for primary research. Studies were identified as positive when clearly addressing the issues, biases, generalization, methods of data collection, and analysis. Neutral and negative studies were indicated as neither strong or weak, and not all issues were adequately addressed. For non-RCTs, risk of bias (RoB) was assessed by either RoB in non-randomised studies of interventions (ROBINS-I) or ROBINS of exposure [32]. Meta-analysis Of Observational Studies in Epidemiology (MOOSE) group was recommended by previous review [33]. 

Nutritional adequacy was determined by comparing the recommended daily dietary requirement with the individual or average population actual intake. The analysis of nutritional adequacy is valuable in determining the risk of deficiency and correlation with disease [34]. Studies may use recommended dietary allowance (DRI), estimated average requirement (EAR) or adequate intake (AI) as reference for recommended dietary intake (RDI) comparing to patient intake. Referencing RDI varied across studies as the nutrients are reviewed every few years and are country-specific depending on the cultural diet. 

## 3. Results

**Figure 1:**
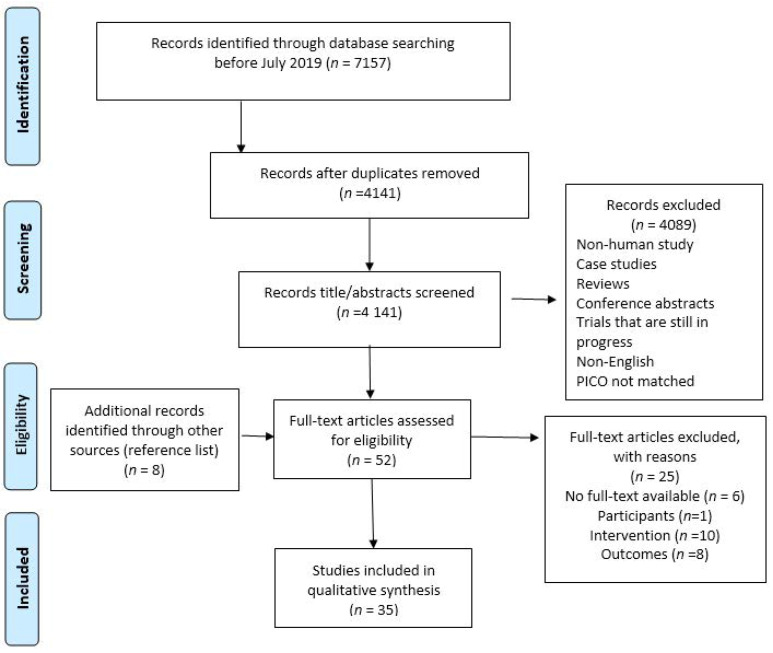
PRISMA flow chart diagram of study selection process. Note. Lab studies without human participants (participants), studies without texture-modified diets (TMDs) or thickened fluids (TFs) consumers (intervention) or study outcomes did not include nutrition measurement (outcomes) were excluded.

A total of 35 studies were considered as eligible for final analysis following PICO criteria. Figure 1. demonstrates the evaluation and selection process using 2009 PRISMA guideline for systematic reviews: 4141 non-duplicated studies were identified for initial title and abstracts screening, full texts were retracted for 52 articles, and 8 additional relevant studies were manually identified from reference lists. 

Table 1 shows the participant characteristics and study outcomes. Summarised study designs of eligible studies is categorized in Table S2 (Supplementary). Studies were conducted in hospitals (*n* = 14), LTCs (long-term cares) (*n* = 18) or a combination of both (*n* = 3) across 12 countries. The number of studies investigated the nutrition outcomes of TMDs/TFs increased since 1991 (Figure S1, Supplementary). Observational studies evaluated the nutrition intake and menu between TMDs and regular diets contributed to one-third of the publications. An increase in the number of studies focusing on shaped or moulded TMDs was found in the last 10 years. The average age of the totaled 2245 patients (883 and 1362 from experimental and observational studies respectively) ranged from 57 to 89 years old. 4 studies examined the nutrient provision of LTC or hospital menu included 135 diet order of TMDs (*n* = 68) and regular diet (*n* = 67) [35,36,37,38]. 

The descriptors of TMDs and TFs varied across studies, 3 studies reported with previously used UK descriptors: Texture B, C, D, E for TMDs, and Stage 1, 2, 3 of TFs [14,63,66], while Miles et al. used IDDSI terminology. Pureed was the most common TMD studied [19]. Other TMDs included homogenised, blended, minced, minced/pureed, chopped, soft and soft/minced. TFs were named as honey-, nectar-, pudding-like; mildly thick and moderately thick viscosity

### 3.1. Nutrition Intake

Twenty-five studies assessed nutrition intake. Reyes-Torres et al. used 24 h recalls, Keller et al. and Nowson et al. used validated visual estimation methods of plate wastage [49,54,57]. The other 23 studies measured nutrition intake via weighed food record and/or clinical dietary record (Table S3, Supplementary).

#### 3.1.1. TMDs and Regular Diet

Studies had similar exposure, and low risk of bias, therefore, the data were pooled. Pooled data from 712 patients demonstrated that overall, both energy and calcium intakes were in favour of the regular diet, with a significant mean difference of 237.9 kcal/day (95%CI: 140.8 to 321.0, *p* < 0.00001) and 63.1 mg/day (95%CI: 49.3 to 77.0, *p* < 0.00001) respectively (Figures S2 and S3, Supplementary S3). Whereas, protein intake did not significantly vary (6.3 g/day, 95%CI: −0.81 to 13.45, *p* = 0.08) between diets (Figure S4, Supplementary). Risk of bias from observational studies included in meta-analysis was listed in Table S2 (Supplementary). 

Energy and protein intake for patients consuming cook-fresh TMDs ranged from 908.2 ± 47.8 to 1764.3 ± 283.2 kcal/day and 39.5 ± 1.9 to 69.7 ±10.2 g/d established by Sherwin et al. and Massoulard et al. respectively [51,59]. Out of 6 studies comparing energy and protein intake between TMD and regular diet consumers, 4 showed significant higher energy intake consuming regular diet, whereas only 2 studies found significant difference in protein intake. 

Foley et al. demonstrated there were no significant differences in either energy or protein intakes between TMDs and regular diet among acute stroke patients within 21-day period [43]. A higher energy intake was achieved by those receiving supplements in Wright et al. study [40]. Comparing to regular diet, the intake of non-starch polysaccharide (NSP) [14], fibre [54,59], and fat [51] were significantly lower in TMD consumers. Female older adults consuming pureed diet had a significantly lower intake of iron, thiamin, and vitamin B6, but a higher vitamin C intake compared to regular diet [47]. 

#### 3.1.2. Thickened Fluids 

When accounting for both foods and fluids, fluid intake was significantly lower in patients on TMD and Level 0: Thin fluid consumers compared to those on regular diets (IDDSI Level 1–7) [14] and also in TMD with TF consumers compared to those with enteral or parenteral feeding [62]. Six studies investigating TF intake measured outcomes of hospital patients. Total fluid intake varied greatly without quantifying solids and external fluids, ranging from 278 ± 233 [65] to 1428 ± 7 mL/d [48]. Free water protocols had varying results. There was no significant difference in total fluid intake when stroke patients on TFs were allowed access to water, but TFs intake was significantly lower in the water access group [44]. However, in another 8-day RCT, a significantly higher fluid intake was found when patients were allowed water [48]. Both studies indicated the amount of water consumption did not exceed TF intake. Provision of pre-thickened fluid significantly improved fluid intake in non-acute patients [65]. However, a significantly higher energy, protein, calcium, vitamin C, and vitamin D intake from drinks was found when patients were given pre-thickened fluids compared to powder-thickened fluids [52]. All 3 studies indicated hospital patients on TFs had inadequate fluid intake compared to the requirement of 30 mL/kg BW/day [42,62,65]. Patients on supplementary fluids or use the combination of TFs and supplementary fluids were able to achieve their fluid requirements [42,62]. Whelan proved the pre-thickened fruit drinks contain higher energy compared to powder-thickened (372 vs. 289 kJ/100 mL) [65].

#### 3.1.3. Texture and/or Nutrition Enhanced TMDs and Traditional Cook-Fresh TMDs

Traditional TMDs are used to describe the cook-fresh TMDs without nutrition or texture modification. Though 8 studies indicated an improvement in energy intake using modified TMDs, the improvement was only significant when enzyme-infused TMDs were shaped to appear like regular-food [46]. Welch and colleagues used fibre-fortified cereals and oral nutrition supplement (ONS) in pureed diet and Ott et al. implemented shaped and whey protein-fortified TMDs [55,64]. Data of energy and protein intake was pooled from 3 studies that evaluated the intervention of shaped TMDs [45,46,57]. Meta-analysis demonstrated that overall, both energy and protein intake were in favour of shaped TMDs, with a significant mean difference of 273.8 kcal/day (95%CI: −419.1 to −128.6, *p* = 0.0002) and 12.4 g/d (95%CI: −17.9 to −6.8, *p* < 0.0001) (Figures S5 and S6, Supplementary). 

Shaped TMD consumers had a significantly higher fat intake, though no significant differences were found in carbohydrates and fibre intake [45,49]. Significantly higher fluid intake was found in meal patterns with small and frequent meals [60]. 

Although fortified infant cereal-fortified TFs demonstrated an increased micronutrient intake, no statistical tests were performed [56]. Welch et al. demonstrated that pureed diet consumers had significantly higher intake of magnesium, phosphate, calcium, iron, zinc, and 9 vitamins (vitamin B1, B2, B3, B6, B12, vitamin A, vitamin C, vitamin D, and vitamin E) with ONS and fibre-fortified cereals in comparison to consuming traditional pureed diet alone [64]. Intakes for all investigated vitamins increased significantly with vitamin powder fortification [35]. Consumption of shaped TMDs also showed significantly higher intake in 5 micronutrients (potassium, magnesium, zinc, vitamin B3, and vitamin D) [45].

### 3.2. Meal Consumption

#### 3.2.1. TMDs and Regular Diet

Table 2 summarises the studies that measured meal consumption. Few hospital patients (*n* = 4) on TMDs were able to complete a full meal [66]. Among soft, blend, and regular diet, de Sá et al. observed patients consuming soft diet had the lowest consumption of main meals, but the highest snack consumption [39]. And, Miles et al. found LTC residents consuming pureed diet were more likely to consume the full meal [19].

#### 3.2.2. Texture and/or Nutrition Enhanced and Traditional Cook-Fresh TMDs

Though an increase in meal consumption was observed in all 7 studies using modified TMDs compared to traditional TMDs, only Torrence reported a significant effect with shaped TMDs (*p* < 0.05) [61]. Zanini et al. demonstrated a high compliance of meal consumption after texture-individualised modification [67]. Decreased main meal consumption was reported when combined with the provision of ONS [39]. ONS was better accepted in the afternoon for both soft and blended diets, 96.8% and 100% respectively, though no statistical tests were reported.

Farrer et al. surveyed the reasons for food wastage [41]. Clinical reasons associated with swallowing or disease were reported as the most common reason for food wastage, 81% of the moulded pureed group and vs. 84% of the control group with traditional pureed diet. Others chose the reason of ‘food issues’ representing dislike or not enjoying the meals. Positive effects on plate wastage were found in moulded pureed group, but was not significantly less (*p* = 0.09).

### 3.3. Nutrition Adequacy

#### 3.3.1. TMDs and Regular Diet

Nutritional adequacy was determined by comparing patient intake with their DRVs. Dietary recommendation reference varied among studies by years and countries as shown in Table S4 (Supplementary). Though regular diet consumers showed a significantly higher percentage of adequate energy and protein intake [54], neither TMD nor regular diet consumers in LTCs met the energy recommendations [14,47,51]. Both were able to meet their protein requirement. Foley et al. reported a majority of the acute stroke patients (75.7–94.4%) were able to meet the energy requirement with the consumption of TMDs or regular diet during the 21-day admission, but inadequate intake of protein [43]. 

NSP and fibre intake were less than the recommendations in both diets [14,54]. A high risk of inadequate fluid intake with pureed diet was found by Philip and Greenwood [56]. Average fluid intake from TMD consumers met 75% of the estimated fluid requirement, which was significantly less than the lower limit [14]. 

Of 17 micronutrients assessed with TMD consumptions from 5 studies, 65% (iron, zinc, calcium, magnesium, vitamin B1, B6, B12, vitamin D, vitamin E, folacin and pantothenic acid) were unable to meet the dietary requirements [35,47,54,56,64]. Consumption of hospital TMDs had a significantly higher prevalence of inadequate intake in sodium, iron, zinc, calcium, and manganese compared to regular diet [39]. 

#### 3.3.2. Texture and/or Nutrition Enhanced TMDs and Traditional Cook-Fresh TMDs

Studies showed both hospital and LTC consumers had inadequate energy intake with traditional TMDs, except Philip and Greenwood [56], who reported mixed results. Germain et al. and Higashiguchi indicated patients still failed to meet their energy requirement with shaped TMDs [45,46], other studies found adequate intake was achieved with both shaped TMDs and nutrient enrichment [55,56,64]. With modified TMDs, patients reached protein intake recommendations and have a lower risk of inadequate intake. Vitamin-fortified pureed diet achieved a significantly higher prevalence of adequate vitamin intakes and was below the upper limit (UL) in comparison to un-fortified pureed diet, though none of the patients met the recommended vitamin D level [35]. Philip and Greenwood also found infant cereal-fortified pureed diet decreased the risks of inadequate intake except for folate and fluid [56]. Besides calcium, no significant improvements in the prevalence of adequate mineral intake were found with the provision of ONS in hospital patients [39]. On the other hand, LTC residents consuming commercial supplements and fibre-fortified cereal with TMDs were able to achieve 100% of their micronutrient recommendations after 3 and 6 months [64]. Overall, nutrient-enriched TMDs lowered the risk of inadequate intake in all micronutrients assessed. 

### 3.4. Nutrition Content of the Meal

#### 3.4.1. TMDs and Regular Diet

All 4 studies found the level of energy, protein, carbohydrates, fat, and fibre content were all lower in TMD menu compared to a regular diet menu, though only Durant proved the significance [40]. Regular diets offered significantly richer micronutrients (iron, zinc, vitamin E, vitamin K, pantothenic acid), but less vitamin D compared to pureed diet [63]. Pureed menu in LTCs was less likely to provide adequate nutrients, in particular, fibre, zinc, potassium, calcium, magnesium, vitamin B6, vitamin D, vitamin E, vitamin K, and folacin [36,47,63]. 

Both TMDs and regular diets provided adequate vegetable serves, but inadequate protein or carbohydrate portions on the plate, particularly TMDs [19]. de Sa et al. compared the ONS provided in soft diet against regular and blended diet [39]. ONS contained high potassium (3155 mg, 2615 mg) in both diets and high calcium in regular and blend diets (710 mg), but without phosphorus and sodium. Trace elements (iron, zinc, copper, manganese, and selenium) provided by hospital regular and TMDs with or without ONS were both insufficient to comply with dietary recommendations in most occasions, and the values varied across months [53]. 

#### 3.4.2. Texture and/or Nutrition Enhanced TMDs and Traditional TMDs 

There were no significant differences in energy and fluid offered between 3 regular TMD meals with small and frequent TMD meals, significance of nutrient content before and after experiment adjustment was only reported by Taylor and Barr [60]. Modified TMDs offered sufficient nutrients [46,56,64]. Fortifying TFs or TMDs with infant cereal enhanced the multiple nutrient contents (protein, calcium, iron, vitamin B1, B2, B3) [50,56]. Pre-prepared and enzyme-infused shaped TMDs contained equal or denser nutrients compared to traditional TMDs [46,49].

### 3.5. Texture and Consistency

Outcomes of meal texture and consistency are shown in Table 3. No suitable snacks were provided for TMDs in LTCs [14,19]. Rosenvinge and Starke evaluated the compliance of TMDs and TFs pre- and post-staff education [58]. Provision of inappropriate consistency of TFs was the most common reason for the non-compliance (69.8%), and 25% of the time the drink was not thickened. Not all meal components were modified or to the appropriate levels. Texture and consistency of pureed foods improved after fortification with infant cereal or shaping with thickener [37,50].

### 3.6. Quality Assessment 

Over half of the studies were rated as ‘neutral’ quality (*n* = 24), and 10 were rated as ‘positive’. Main contributors to the ‘neutral’ quality were due to the small number of patients who were not representative, and randomisation or blinding was not used. Sample size largely varied, from 10 [35] to 479 [67]. Only Kennewell and Kikknakos was rated as ‘low’ due to a lack of explanation of plate waste measurement tool [50]. Despite the method of visual estimation being less accurate compared to weighing on scale, the method was validated in the settings. Nutrition value was calculated via a variety of software options, dietary information relied on clinical staff observations and reports may be less accurate. All studies reported their limitations except de Sa et al. [39]. 2 trial studies were not able to demonstrate statistical significance due to small sample sizes [41,50]. A funnel plot was generated for meta-analysis; asymmetric plots were found in experimental studies assessing protein intake representing potential publication bias. The plot was towards the top indicating a lack of smaller trial publications [68]. Larger trials with positive results and English studies were more likely to be published and cited, however, it is possible that the heterogeneity in study methods and clinical settings may lead to asymmetry.

## 4. Discussion

The aim of this systematic review and meta-analysis was to investigate the strength of evidence for the use of TMDs on nutrition-related outcomes for adults with dysphagia. This is the first review of both nutrition intake and meal content across the range of TMDs. Thirty-five studies, incorporating 1455 LTC residents (average age ranged from 58 to 87 yr), 651 hospital patients (average age ranged from 57 to 82 yr) and 139 mixed LTC and hospital participants (average age ranged from 67 to 87 yr), met the inclusion criteria conducted across 12 different countries. Intervention duration ranged from 7 days to 12 months among 17 experimental studies. Evidence highlights poor energy and fluid intake with insufficient nutrients offered from TMDs and supplements, leading to a higher risk of nutrition inadequacy. Reports of inappropriate texture and consistency of foods and drinks were also concerning. Results suggest improved nutrition intake in both macro and micronutrients is achievable by modifying TMDs either visually, textually, via customization, through meal fortification or providing ONS. The strongest evidence currently is modification TMDs by shaping/moulding TMDs into regular-food liked shape and enhancing the consistency, which achieved a 32–36% improved protein intake [37,45]. Fortification also contributes to lower rates of nutrition inadequacy. 

### 4.1. Nutrition Intake and Meal Consumption

Reporting errors should be considered with the studies relying on clinical records or 24 h recalls due to the subjective nature of reporting and the dependency of participant memory in an older adult group [69]. Validated visual estimation of plate wastage should be administrated by trained staff when weighed plate wastage is not achievable or investigating a greater population [59]. Within the 20 studies assessing the oral food intake, 11 included the snacks, 8 included ONS. Although collecting foods and drinks outside the mealtime is challenging, this may contribute significantly to the overall intake and meal patterns [39]. Minimal suitable snacks were offered to TMD consumers in LTCs, which provided significantly less energy and NSP intake in TMD consumers [14,19]. The pureed diet contained less fibre content compared to regular diet [63]. This may be due to the avoidance of ‘high risk’ foods of TMDs, such as fibrous textured fruits and vegetables, husks, and bread, therefore contributing to the reduced NSP and fibre intake [14,54,59]. The insufficient fibre and fluid intakes are associated with a higher risk of constipation [70]. While Welch et al. implemented the fibre-fortified cereal with a pureed diet, the consumers still had a low intake [64]. Efficient fibre-enrichment options should be investigated in future studies.

The assessment of micronutrient intake has been less of a focus in the current literature. Nutrients at higher risk of deficiency in older adults such as iron, zinc, calcium, vitamin B6, vitamin D [71,72,73] were found to be inadequate [39,47,54,59]. Fortification and ONS demonstrated the ability to improve micronutrient intake in LTC pureed diet consumers [35,56,64]. Older pureed diet consumers in LTC appeared to have a reasonable acceptance of oral supplements [64]. However, compliance is associated with the severity of the clinical condition evidenced by poor compliance of the ONS in hospital dysphagic patients [39,66,74]. Prescribed intake and exact quantities of ONS were not described in the studies. More studies are required to conclude whether providing ONS contributes to a higher nutrition intake with TMDs.

Previous reviews suggest older adults consume a higher energy intake with small and nutrient-dense foods [75], though TMD consumers only reported an increased fluid intake with small and frequent meals when comparing to three-meal pattern [60]. 

Although meal consumption is a good reflection of patient acceptance of the meal, food quality, feeding assistance, and mealtime environment also contribute to nutritional intake [76]. It is uncertain whether increased intake is due to higher meal consumptions or improved nutrient density of the meals. The only studies reported both nutrition intake and meal consumption changes [46,49], neither reported significant changes in meal consumption with the 3-day or 6-month intervention. However, energy and protein intake significantly improved while a significantly less food was consumed with enzyme-infused shaped TMDs [46]. Study duration of the experimental studies investigated meal consumption were all under 16-day, except Keller et al. [49]. The results may be impacted by the novelty change. Future studies should consider examining influence of meal consumption on nutrition intake and nutritional status in longer intervention period.

Nine studies assessing fluid intake were unable to be compared due to differing criteria of total fluid intake. Two studies took the total measurement of fluids and foods [14,62], whereas water content of solid foods was not accounted in other studies. A higher intake was observed when TFs consumers had access to water or external fluids (enteral/parenteral feeds or IV fluids). Fluid provision and intake should be monitored in future research, to confirm the effective strategies before firm recommendations could be made.

### 4.2. Nutrition Content

Pureed meals appeared to have lower nutrient-density compared to regular diets in LTCs [36,38,40,47,63]. It appears that although the meals offered sufficient nutrients, TMD consumers were still unable to meet their requirements or were at risk of inadequate intake, given the inability of completing their meals [46,47,54,56]. TFs and hospital TMDs were under-investigated. Freshly made TMDs offering inconsistent nutrient levels were reported by both Moreira et al. and Dahl [38,53]. Purchasing pre-made high energy-density texture-modified meals may be beneficial for hospitalised older people [77]. 

### 4.3. Meal Texture 

Appropriate texture and consistency play an important role in ensuring patient safety and care [78,79]. The lack of terms and descriptions of texture and consistency modification was considered a compliance challenge. In the 5 studies assessed, the meal texture was inconsistent [14,19,37,50,58]. Variance in appearance and texture was observed in moulded vegetables, and therefore was not included in the intervention [41]. Inconsistency in the production of texture modified food may also worsen the patient meal experience [15] and limit their food consumption. Modification with thickener or enrichment enable better texture and consistency [37,50], inappropriate use of food thickeners could lead to the change of consistency. 

### 4.4. Study Implications

Despite pooling relevant data, there was variance in TMD descriptors as expected in a systematic review of the past literature. In 2013, Cichero et al. reviewed TMD terminologies across countries during the development of the IDDSI framework [80]. Considerable variation existed in the texture modification and descriptions used. Though pureed diet has been extensively studied, the lack of description and testing limits interpretation of these older study findings against the new standard IDDSI Level 4: Puree. Due to this challenging terminology mapping, there is insufficient overlap in classifications to warrant the pooling of data. Although IDDSI provides an instructed audit tool and specification for each level of TMDs and TFs, it is still a recently introduced framework and requiring more work on validating the testing methods [81]. To minimize the confusion in terminologies and provide quantitative comparisons, robust study designs with detailed description of categorization and testing methods are required to study the various levels of TMDs and TFs. Future studies should consider using validated tools and international references for comparison. For example, nutritional status can be assessed with Mini-Nutritional Assessment—Short Form (MNA-SF). Biomarkers such as c-reactive protein, albumin, and transferrin can also be used as indicators of nutritional status in older adults. Evaluation of functional and clinical changes should be considered in future studies, as very few studies incorporated these. 

Due to the mixed population and variance in participant medical conditions, it is difficult to draw conclusions about nutritional intake and adequacy. Only a few studies assessed the cognitive status, physical function, and self-feeding ability, which are noteworthy effects on the progress of dysphagia and nutritional outcomes [82]. Future research may consider using multidisciplinary intervention and assess the etiologies of compromised nutritional intake in different settings [83]. 

Though only 5 of the included studies were RCT design, more trials using shaping/moulding TMDs are needed. The majority of the studies had a neutral quality rating with a number compromised by a small number of patients. Large multi-center trials are in demand to overcome the diversity in participants and foodservice. In view of the challenges in eating adequate quantities of TMDs, more attention needs to be paid in enhancing the flavor and assessing the texture and consistency of the foods to ensure patient safety. Intense flavour and taste are key contributors to patient enjoyment of TMDs [84]. Meanwhile, researchers should consider the possible consistency alternation with the use of thickener or shaping agents during the shaping/moulding process, which may result in a change of TMD level and sensory aspects [12]. TMDs should be audited regularly against descriptors.

## 5. Conclusions

Optimal nutrition care strategies based on evidence are required for the management of dysphagia. Promising improvements in nutrition intake were achievable through modifying meal texture and consistency (using enzyme-infusion shaping, 3D moulding, consistency-control), fortification (infant-cereal fortification in pureed or TFs, and vitamin fortification powder, whey protein, and rapeseed oil fortification) and offering ONS. Screening and enhancing TMD menus are required in both acute and long-term cares. Creating and offering nutrition-dense and texture-appropriate TMDs would be beneficial for older patients with dysphagia. A multidisciplinary approach should be incorporated in future research. Dysphagia and malnutrition screening should be implemented as part of standard nutrition assessment in hospitals and LTCs. To achieve the best nutritional care, multidisciplinary approach should be adapted. Dietitians and speech-language therapists should share knowledge and support foodservice and care staff in recipe development, monitoring food and clinical signs, targeted feeding training. Clinicians should also actively involve patients and family to adhere to the treatment plan. Future research should also consider investigating available clinical approaches to assist the improvement of nutrition status by including education programs, by making adjustments to dining environments, and by offering feeding assistance and social support.

## Figures and Tables

**Table 1:** Characteristics of included studies in systematic review [14,19,35,36,37,39,40,41,42,43,44,45,46,47,48,49,50,51,52,53,54,55,56,57,58,59,60,61,62,63,64,65,66,67,68].

Source	Method	Setting, Origin	Patient Characteristics	Interventions	Comparator/Control	Outcomes	Quality Assessment
Adolphe et al. (2009) [35]	Pre-post Experimental 8 weeks	LTC Canada	Residents ≥ 50 y consumed pureed diet daily Ex: Palliative Mean BMI (kg/m^2^) 25	Vitamin-fortified pureed diets (lunch, dinner) *n* = 10	Unfortified pureed diet *n* = 10	-Vitamin intake (3-day weighed food record) -Adequacy (USA 2003)	Neutral
Bannerman and McDermott (2011) [14]	Observational Cross-sectional	3 LTCs Scotland	Residents >60 y Ex: Nil by mouth, receiving artificial nutritional support, fluid restriction, acutely unwell, palliative Mean age (y) 88.1 ± 5.4	Texture C–Thicker pureed: *n* = 11 Texture D–Minced/moist: *n* = 4 [UK national descriptors 2009]	Regular diet *n* = 15	-Macronutrient and fluid intake (3-day weighed food record) -Adequacy (UK 1991) -Meal compliance -Role of snacks Snacks and fluid included	Neutral
Beck and Hansen (2010) [36]	Observational Cross-sectional	Meals-On-Wheels and LTCs Denmark	Diet orders from 10 Kitchens preparing meals for LTCs and Meals-On-Wheels	Chopped diet *n* = 9 Blended diet *n* = 6	Regular diet *n* = 10	-Nutrient content	Neutral
Cassen et al. (1996) [37]	Pre-post Experimental 16 days	LTC US	All residents consumed pureed diet Ex: discharged or passed away	3D shaped pureed diet *n* = 18	Unmodified pureed diet *n* = 18	-Energy and protein intake (clinical record) -Meal consumption	Neutral
Dahl et al. (2007) [38]	Observational Cross-sectional	8 LTCs Canada	Residents consumed pureed diet	None	Pureed diet *n* = 20	-Energy and protein intake (3 to 5-day weighed food record) -Adequacy (USA 2003) -Nutrient content ONS and snacks included	Neutral
De Sa et al. (2014) [39]	Observational Cross-sectional	Oncology hospital Brazil	Oncology patients (admitted for surgery *n* = 83, intercurrence *n* = 58, chemotherapy *n* = 21, radiotherapy *n* = 1), mean stay 6.3 ±1.2 days) Mean age (y) 57 ± 15	Regular + OFC *n* = 29 Soft + OFC *n* = 4 Blend + OFC *n* = 8	Regular *n* = 97 Soft *n* = 6 Blend *n* = 19	-Mineral adequacy (snacks included) -Meal consumption (plate wastage) -Supplement acceptance and content Snacks included	Neutral
Durant (2008) [40]	Observational Cross-sectional	LTC Canada	Diet orders from regular or pureed diets over 5-week menu	Pureed with thin fluids *n* = 9	Regular diet *n* = 19	-Nutrition content (duplicate meal trays) ONS excluded	Neutral
Farrer et al. (2016) [41]	Pre-post Experimental 2 weeks	Hospital Australia	All patients > 18 y consuming pureed diet in any acute care units (Cancer, *n* = 17, Parkinson *n* = 3, Cerebral palsy *n* = 3, Respiratory *n* = 10, Head trauma *n* = 5, Other *n* = 27) (reasons for pureed diet: dysphagia *n* = 40, comfort *n* = 6, temporary swallowing difficulty *n* = 18) 70% ≥ 65 y	Moulded pureed diet (Texture C) *n* = 27	Unmodified pureed diet (Texture C) *n* = 38	-Meal consumption (plate wastage)	Neutral
Finestone et al. (2001) [42]	Observational Cohort 21 days	Hospital Canada	Dysphagic patients admitted to hospital within 5-days of onset of first stroke (evaluated by SLT) Mean age (y) 66.1 ± 13.5	Dysphagia diets (chopped/minced/pureed) + TF *n* = 6	Started with enteral feed/IV fluids, progress to dysphagia diet +TF after 7–9 days *n* = 7	-Fluid intake (2-day oral food and fluid intake record/5-day fluid balance sheets of enteral feed) -Fluid Adequacy (PENG 2004) Solid excluded	Positive
Foley et al. (2006) [43]	Observational Cohort 21 days (data collection at day 1, 7, 11, 14, 21)	Hospital neurological unit UK	Well-nourished acute stroke patients (evaluated by SLT) Mean age (y) 69 ±11.3	Dysphagia diets *n* = 11–20	Regular diet *n* = 25–48 Enteral feed *n* = 11–20	-Nutrition intake (2-day of calorie counts by portion for oral diets and fluid balance sheets for enteral feed) -Adequacy (indirect calorimetry)	Neutral
Garon et al. (1997) [44]	RCT 1 year	Hospital stroke rehabilitation UK	Stroke patients with previously identified thin fluid aspiration by videofluoroscopy Mean age (y) 76.8	TFs + free access of water *n* = 10	TFs only *n* = 10	-Fluid intake (Daily flowcharts) Solid excluded	Positive
Germain et al. (2006) [45]	RCT 12 weeks	LTC Canada	Residents aged 65–90 y admitted ≥3 m and had >7.5% weight loss in the last 3 m or BMI < 24 with dysphagia evaluated by RIC tool (Alzheimer *n* = 8, Dementia *n* = 6, Stroke =2, Parkinson *n* = 1) Ex. Cancer, chronic intestinal disease, terminally ill patients Mean age (y) 59	Shaped minced, minced/pureed or pureed diet and consistency-controlled TFs using Bostwick consistometer (nectar, honey, pudding) *n* = 9	Unmodified minced−70, minced−3 or pureed diet and honey level TF (consistency not systematically controlled) *n* = 8	- Macro- and micronutrient intake (2-day weighed food record) -Adequacy Snacks and ONS included	Neutral
Higashiguchi (2013) [46]	Experimental Cohort 7 days	17 hospital/LTC Japan	Inpatient with mastication difficulty under nutritional management and residents on TMDs with inadequate consumption (Stroke *n* = 19, Cancer *n* = 9, Heart failure *n* = 7, Fracture *n* = 5, Dehydration *n* = 4, Pressure ulcers, *n* = 3, Pneumonia *n* = 2, Anemia *n* = 2, COPD *n* = 2, Dementia *n* = 2, Diabetes *n* = 1, Parkinson *n* = 1, Other *n* = 17, None *n* = 2) (require total meal assistance *n* = 17, partial *n* = 6, none =34) Mean age (y) 81.6 ± 9.3 Mean BMI (kg/m^2^) 18.8 ± 0.34	3 days of nutrient -dense (Enzyme-infused) TMDs (enzyme was evenly infused into the ingredients to adjust the homogeneous softness of the meal instead of adding water for softening) Nutrients were not diluted and volume not increased *n* = 55	4 days of unmodified TMDs	-Macronutrient and sodium intake (mean meals weighed food record) -Meal consumption -Adequacy -Nutrient content (weighed plate wastage) Mean weight of the modified food is lower than unmodified	Positive
Johnson et al. (1995) [47]	Observational Cross-sectional	LTC US	70 randomly selected female residents ≥65 y admitted for ≥6 m consuming food orally Mean age (y) 85	Pureed, *n* = 20	Regular diet, *n* = 31	- Macro- and micronutrient intake (7-day food record by consumption monitoring system) -Adequacy (US 1989) -Nutrition content Snacks and ONS included	Neutral
Karagiannis et al. (2011) [48]	RCT 8 days	Hospital subacute units Australia	Patients ≥18 y aspirated on thin liquids with prescription of modified or TF diet by SLTs without chronic respiratory conditions or prior tracheostomy (Stroke *n* = 40, Dementia *n* = 11, Alzheimer *n* = 7, Parkinson *n* = 5, Cancer *n* = 10, Motor neuro disease *n* = 1, Huntington *n* = 1, accident *n* = 1) Mean age (y) 79.5	TMDs (Pureed; Minced; Soft/Minced) + TF (Honey; Pudding; Nectar) + free access of water *n* = 42	TMDs + TF *n* = 34	-Fluid intake (Total daily oral liquid record) Solid excluded	Positive
Keller et al. (2012) [49]	Pre-post Experimental 9 m	Hospital and LTC Canada	All dysphagic residents fully consumed pureed or minced diets (Stroke, Parkinson, Dementia) Ex. enteral feed Facility avg. age 67 and 82 yr	6 m of mix of 61% bulk and 39% shaped ready-to-use (reduced nutrients dilution and easier to chew and swallow) commercial TMDs *n* = 40	3 m of bulk commercial TMDs (unshaped, packaged in bulk) *n* = 40	- Macro- and micronutrient intake (4 and 6-day of lunch/dinner food record by estimation) -Meal consumption (visual estimation of plate wastage) -Nutrient content Snacks and ONS excluded	Positive
Kennewell and Kokkinakos (2007) [50]	Observational Cross-sectional	2 hospitals Australia	Dysphagic patients (unspecified aetiology)	Infant-cereal fortified minced/pureed diets	Unfortified minced/pureed diets	-Iron content -Meal consumption (plate wastage) -Meal compliance	Low
Massoulard et al. (2011) [51]	Observational Cross-sectional	4 LTCs France	All residents with chewing and/or swallowing difficulties Mean age (y) 85.8 ± 9.3 Mean BMI (kg/m^2^) 26.8 ± 6.5	Chopped *n* = 12 Mixed *n* = 26	Regular diet *n* = 49	-Nutrition intake (24 h weighed food record or staff reported survey) -Adequacy (France 2009)	Neutral
McCormick et al. (2008) [52]	Crossover Cohort 12 weeks	Geriatric hospital UK	Dysphagic patients who had were identified as at risk of aspiration and require TFs Mean age (y) 76 Mean BMI (kg/m^2^) 23.3 ± 0.4	6 weeks of pre-thickened TFs *n* = 11	6 weeks powder-thickened TFs (modified maize starch) *n* = 11	-Macro- and micronutrient, fluid intake (Total daily oral liquid record) -Fluid adequacy (PENG 2004) Solid excluded	Positive
Miles, Liang et al. (2019) [19]	Observational Cross-sectional	10 LTCs New Zealand	All residents ate in the dining room on the data collection day Ex. Residents ate outside the dining room Mean age (y) 78	Pureed, *n* = 101 Minced and Moist, *n* = 99 Soft and Bite-sized, *n* = 100 [IDDSI, 2018]	Regular diet, *n* = 100	-Meal consumption (visual estimation) -Nutrition content -Meal compliance -Snacks & Fortification	Neutral
Moreira et al. (2014) [53]	Observational Cross-sectional	Hospital Brazil	Diet orders from oncology patients	Blend, *n* = 6 Soft, *n* = 6	Regular diet, *n* = 6	-Supplement content	Neutral
Nowson et al. (2003) [54]	Observational Cross-sectional	14 wards/dining rooms from LTCs Australia	Residents ate in the dining room on the data collection day Mean age (y) 82.9 ±9.5	Pureed, *n* = 53 Soft/Minced, *n* = 48	Regular diet, *n* = 114	-Macro- and micronutrient intake (1-day food record by visual estimation of plate wastage) -Adequacy (Australia 1991) Snacks excluded	Neutral
Ott et al. (2019) [55]	Pre-post Experimental 12 weeks	2 LTCs Germany	Residents diagnosed with chewing and/or swallowing receiving TMDs regularly (all patients had cognition impairment) Mean age (y) 86.5 ± 7.4	6 weeks of single level of reshaped TMDs and enriched with 600 kcal energy and 30 g protein *n* = 16	6 weeks of usual TMDs (completely pureed or partial soft food) *n* = 16	-Energy and protein intake (3-day weighed food record) -Adequacy (German 2015) Snacks and ONS included	Neutral
Philip and Greenwood (2000) [56]	Observational Cross-sectional	Chronic-care hospital and LTC Canada	Residents/patients ≥65 y consumed ≥ 2 pureed entrees and/or TFs Ex: mixed texture diets, energy-controlled diets, specialized diets Mean age (y) 87.1 Mean BMI (kg/m^2^) 21.0	Infant cereal fortified TFs with pureed diet *n* = 21	Unfortified TFs with pureed diet Thin fluid with pureed diet *n* = 23	-Macro-, micronutrient and fluid intake (7-day weighed food record) -Adequacy (USA 1989) -Nutrient content Snacks and fluid included	Neutral
Reyes-Torres et al. (2019) [57]	RCT 12 weeks	National institute Brazil	≥65 yr with a caregiver and a confirmed diagnosis of oropharyngeal dysphagia, and consumed TMDs and TFs (evaluated by V-VST and EAT by dietitians) Ex: cancer, kidney/hepatic failure, terminally ill, high risk of aspiration, low oxygen saturation, enteral feed Mean age (y) 76	Consistency modified and standardized TMDs and nectar or pudding level TFs (measured with Brookfield Viscometer) *n* = 20	Unmodified pureed diet with one viscosity of TFs (consistency not systematically controlled) *n* = 20	-Energy and protein intake (24 h recall) -Nutrient content	Positive
Rosenvinge and Starke (2005) [58]	Sequential observational with pre-post intervention	Hospital UK	Dysphagic patients from stroke, medicine, surgical, or geriatric wards (identified by SLTs)	TMDs + Pre-thickened TFs with education, *n* = 39	TMDs + TFs, *n* = 16	-Meal compliance	Neutral
Sherwin et al. (1998) [59]	Observational Cross-sectional	6 LTC Australia	Residents ate in the dining room on the day of collection Mean age (y) 83.8	Soft, *n* = 13 Homogenised, *n* = 26	Regular diet, *n* = 36	-Macronutrient and calcium intake (1-day weighed food record) -Adequacy Snacks excluded	Neutral
Taylor and Barr (2006) [60]	Crossover RCT 8 days	LTC Canada	Residents ≥65 y with dysphagia consumed TMDs (diagnosed by experienced swallowing team) Ex. enteral feed, medically unstable, diabetic diet Mean age (y) 85 ± 6.4 Mean BMI (kg/m^2^) 20.4 ± 3.4	4 days of isocaloric 5 meals pattern of minced/pureed diets *n* = 37	4 days of 3 meals pattern of minced/pureed diets *n* = 37	-Energy and fluid intake (4-day weighed food record) -Nutrient content Snacks and fluids included	Neutral
Torrence (2011) [61]	Pre-post Experimental 15 days	LTC US	All residents consumed pureed diet (6 patients were on nutrition intervention program receiving ONS and/or fortification) Mean age (y) 85 Mean BMI (kg/m^2^) 27.6	Pre-shaped pureed diet *n* = 10	In-house made pureed diet *n* = 10	-Meal consumption at breakfast, dinner and dessert (7.5-day weighed plate wastage)	Positive
Vivanti et al. (2009) [62]	Observational Cross-sectional	Hospital medical & neurosurgical wards Australia	Dysphagic patients consumed TMDs and TFs in for ≥5 days (diagnosed by SLTs) (Stroke *n* = 16, dementia *n* = 12, other *n* = 3) Mean age (y) 74.0 ± 16.2 Mean weight (kg) 62 ± 12.2	TMDs + TFs + enteral/parenteral feed [UK national descriptors, 2002] *n* = 25 (36 days)	TMDs + TFs *n* = 25 (146 days)	-Fluid intake (7-day weighed plate wastage, observation and fluid balance charts) -Fluid Adequacy (PENG 2004) Snacks and fluids included	Neutral
Vucea et al. (2018) [63]	Observational Cross-sectional	32 LTCs Canada	Diet orders from LTCs recruited for M3 study opened for ≥6 m had ≥50 residents over 65 y admitted for ≥1 m	Pureed diet *n* = 32 facilities	Regular diet *n* = 32 facilities	-Macro- and micronutrient content Snacks excluded	Neutral
Welch et al. (1991) [64]	Pre-post Experimental 6 m	LTC US	Residents consumed pureed diet and weighed below average or serum albumin/transferrin levels below normal values (identified from medical records) Mean age (y) 81	Pureed diets with fortified high fiber cereals and commercial supplements *n* = 15	Pureed diets with unfortified cereals *n* = 15	-Macro- and micronutrient intake (3-day food record by staff) -Adequacy (USA 1980) -Supplement/fortification content Snacks and ONS included	Neutral
Whelan (2001) [65]	RTC 14 days	Hospital UK	Acute stroke dysphagic patients required syrup-consistency TFs (diagnosed by SLT or videofluoroscopy) Ex. diagnosis using only a screening tool Mean age (y) 72.3 ± 13.4 Mean weight (kg) 67.7 ± 12.7	Pre-thickened TFs, *n* = 11	Powder-thickened TFs, *n* = 13	-Fluid intake (Total daily oral liquid record and fluid balance chart) -Adequacy (UK) -Nutrient content Solid excluded	Positive
Wright et al. (2005) [66]	Observational Cross-sectional	Hospital elderly and neurology wards UK	All medically stable patients consumed TMDs or regular diet (reasons for TMDs: 80% dysphagia, 20% poor dental state; stroke *n* = 19, fall *n* = 8, other *n* = 3) Ex. < 60 y, nil by mouth, enteral feed, other therapeutic diets Mean age (y) 81.5	Texture B–Smooth pureed, *n* = 10 Texture D- Minced/mashed, *n* = 9 Texture E- Soft, *n* = 11 [UK national descriptors, 2002]	Regular diet, *n* = 25	-Nutrition intake (1-day weighed food record for main meals and food records) -Meal consumption (weighed plate wastage) -Adequacy (Schofieled equation 1985) -Supplements acceptance Snacks and ONS included	Neutral
Zanini et al. (2017) [67]	Pre-post experimental 6 m	20 LTCs Italy	Dysphagic residents > 65 y with low comorbidity levels (diagnosed by a physician or reported in medical records) Ex. high level of comorbidity, medically unstable, terminally ill, chronic or cancer disease, sever dysphagia (DOSS ≥ 2), enteral feed Mean age (y) 79.72 ± 12.31	6 m of personalised levels of density, viscosity, texture and particle size TMDs *n* = 401	6 m of unmodified TMDs *n* = 401	-Nutrient content -Meal consumption (visual estimation)	Positive

Note. Ex.–exclusions; BMI–body mass index LTC-long-term care; y–years old; TMD–texture modified diet; TF–thickened fluids; RIC tool–Rehabilitation Institute of Chicago Clinical Evaluation Dysphagia; SLT–speech language therapist; ONS–oral nutrition supplement.

**Table 2:** Outcome data for studies assessing patient meal consumptions.

Studies	Outcomes
Cassen et al. [37]	15% ↑ intake with 16-day of 3 D moulded pureed intervention
de Sa et al. [39]	Main meal consumption rate: blended–75.3%, soft–74.2% and regular diet–79.7% Main meal with provision of ONS: blended–78.0%, soft–68.9% and regular diet–74.2% Consumption of ONS at morning and afternoon tea: blended–82.6 vs. 100%, soft–84.7 vs. 96.8% and regular diet–81.9 vs. 58.3%
Farrer et al. [41]	↑ %patients consuming from <25% to >75% in 2-week of moulded pureed *p* = 0.03 NS ↓ in plate wastage with moulded compared to non-moulded pureed (160 g vs. 286 g, *p* = 0.09)
Higashiguchi [46]	Enzyme-infused TMDs showed a slightly ↑ consumption compared to unmodified TMDs (69.6% vs. 68.7%, *p* > 0.05)
Keller et al. [49]	NS ↑ by using mix of cMTF and rMTF compared to cMTF (*p* = 0.1)
Kennewell and Kokkinakos [54]	NS ↑ with infant cereal fortified pureed
Miles et al. [19]	% patient consumed the full meal: pureed–59%, minced & moist–55%, soft–52% and regular diet–43%
Nowson et al. [54]	% patient consumed the full meal: pureed–74.1 (19.8) %, soft–76.7 (24.5) % and regular diet–82.2 (16.9)%
Torrence [61]	Significant ↑ consumption of breakfast (*p* = 0.007), dinner (*p* = 0.017) and dessert (*p* = 0.005) with pre-shaped pureed
Wright et al. [66]	13% (*n* = 4) of the patients on TMDs completed the full meal
Zanini et al. [67]	83.4% and 12.3% of the texture-individualised TMDs were fully or partially consumed respectively 4.1% of the meals were not eaten

Note. NS–not significant; ↑–increased; ↓–decrease; ONS–oral nutrition supplement; TMD–texture modified diet; cMTF–commercial bulk buy modified-texture food; rMTF–ready-to-use modified-texture food.

**Table 3:** Outcome data for studies assessing meal texture.

Studies	Meal Compliance Outcomes
Bannerman and McDermott [14]	Unmodified vegetables Unrecognizable meal (mix blend) High-risk foods, e.g., pineapple, tomatoes Mixed consistency, e.g., lumps, crumbs Texture modification was not used on snacks
Cassens et al. [37]	Improved texture of pureed foods after using enhancer and thickener
Kennewell and Kokkinakos [50]	Improved food texture consistency after fortification the pureed foods with fortified infant cereal
Miles et al. [19]	60% (*n* = 6) and 33% (*n* = 1) facilities met IDDSI criteria for pureed and minced and moist diets respectively. None of the facilities provided appropriate soft and bite-sized diet.
Rosenvinge and Starke [58]	TFs consistency sig. ↑ at 2nd audit (64.1% vs. 48.4%, *p* < 0.05) No sig. differences in compliance with dietary modifications (82.5% vs. 78.7%) Sig. ↑ compliance with the quantity of food/fluids was given (35.3% vs. 68.8%, *p* < 0.05), 52.4% of the time patient was fed more than specified
Wright et al. [66]	13% (*n* = 4) patients in TMDs received correct food delivery

Note. IDDSI–International Dysphagia Diet Standardisation Initiative; TF–thickened fluid; Sig.–significant TMD–texture modified diet.

## Supplementary Materials

The following are available online at https://www.mdpi.com/2227-9032/8/4/579/s1, Table S1: search strategy, Table S2: Eligible study catergorisation by study designs, Table S3: Risk of bias of observational studies included in meta-analysis, Table S4: Outcome data for studies assessing nutrition intake, nutrition adequacy, nutrition content of the meals and meal consumption, Figure S1: Eligible studies timeline, Figure S2: Forest plot of the effect of texture modified diets and regular diets on energy intake among older adults, Figure S3: Forest plot of the effect of texture modified diets and regular diets on calcium intake among older adults, Figure S4: Forest plot of the effect of texture modified diets and regular diets on protein intake among older adults, Figure S5: Forest plot of the effect of shaped and traditional texture modified diets on energy intake among older adults, Figure S6: Forest plot of the effect of shaped and traditional texture modified diets on protein intake among older adults.
