# Supplementary material for: Nutritional Intake and Meal Composition of Patients Consuming Texture Modified Diets and Thickened Fluids: A Systematic Review and Meta-Analysis
Source: Healthcare (Basel). 2020 Dec 21;8(4):579. doi: 10.3390/healthcare8040579 (PMC7767351; doi:10.3390/healthcare8040579)
Supplement: Supplementary file 1 [file healthcare-08-00579-s001.pdf]

healthcare

MDPI

Table S1. Search strategy.

**COCHARANE via Central**	1. (texture* adj1 modif* adj (food or foods or diet or diets or meal or meals)).ti,ab,kw.<br>2. ((puree or pureed or mince* or blend* or chop* or soft or soften* or thicken* or liquid* or liquef*) adj1 (food or foods or diet or diets or meal or meals or fluid or fluids)).mp.<br>3. 1 or 2
**MEDLINE (Ovid)**	1. (texture* adj1 modif* adj (food or foods or diet or diets or meal or meals)).ti,ab,kw.<br>2. ((puree or pureed or mince* or blend* or chop* or soft or soften* or thicken* or liquid* or liquef*) adj1 (food or foods or diet or diets or meal or meals or fluid or fluids)).mp.<br>3. 1 or 2<br>4. limit 3 to "humans only (removes records about animals)"<br>5. limit 4 to ("young adult (19 to 24 years)" or "adult (19 to 44 years)" or "young adult and adult (19-24 and 19-44)" or "middle age (45 to 64 years)" or "middle aged (45 plus years)" or "all aged (65 and over)" or "aged (80 and over)")
**EMBASE (Ovid)**	1. (texture* adj1 modif* adj (food or foods or diet or diets or meal or meals)).ti,ab,kw.<br>2. ((puree or pureed or mince* or blend* or chop* or soft or soften* or thicken* or liquid* or liquef*) adj1 (food or foods or diet or diets or meal or meals or fluid or fluids)).mp.<br>3. 1 or 2<br>4. limit 3 to "humans only (removes records about animals)"<br>5. limit 4 to (adult <18 to 64 years> or aged <65+ years>)
**SCOUPUS**	1. TITLE-ABS-KEY ((texture* W/1 modif*) W/1 (food* or diet* or meal*))<br>2. TITLE-ABS-KEY ((puree* or mince* or blend* or chop* or soft or soften* OR thicken* or liquid* or liquef*) W/1 (food* or diet* or meal* or fluid*))<br>3. 1 and 2
**CINAL PLUS (EBSCOhost)**	1. texture* N1 modif* N1 (food* or diet* or meal*<br>2. ((puree* or mince* or blend* or chop* or soft or soften* or thicken* or liquid* or liquef*) N1 (food* or diet* or meal* or fluid*))<br>3. 1 or 2
**Search Results**	*Search date: 5th May, 2019*<br>1. CENTRAL—(Cochrane Central Register of Controlled Trials), 1450 results<br>2. MEDLINE (Ovid), 2313 results.<br>3. EMBASE (Ovid), 2887 results<br>4. SCOPUS, 51 results, 2 more result was found at 25th June, 2019<br>5. CINAL Plus (EBSCOhost), 464 results

healthcare

Table S2. Eligible study categorisation by study designs.

Observational *N* = 18		TMDs compared with regular diet *n* = 13
		TMDs/TFs compared with infant cereal fortified *n* = 2
		TMDs and TFs with/without enteral feed/IV *n* = 2
		Pureed diet without comparison *n* = 1
Experimental *N* = 17	RCT *n* = 5	Use of shaped TMDs *n* = 2
		TFs with/without free water access *n* = 2
		Powder TFs compared with Pre-TFs *n* = 1
	Cross-over *n* = 2	Powder TFs compared with Pre-TFs *n* = 1
		Use of small frequent meal pattern *n* = 1
	Pre-post *n* = 10	Use of shaped/moulded TMDs *n* = 6
		Use of fortified (and shaped) TMDs *n* = 3
		Use of fortified and shaped TMDs *n* = 1
		Education intervention *n* = 1

healthcare

MDPI

Table S3. Risk of bias of observational studies included in meta-analysis.

Study	Risk of Bias						
	**C**	**S**	**ME**	**DE**	**MI**	MO	R
Bannerman and McDermott	Serious	Low	Moderate	Low	Low	Low	Low
Johnson et al.	Serious	Low	Moderate	Low	Low	Moderate	Low
Massoulard et al.	Serious	Low	Moderate	Low	Low	Moderate	Low
Nowson et al.	Serious	Low	Moderate	Low	Low	Moderate	Low
Sherwin et al.	Serious	Low	Moderate	Low	Low	Low	Low
Wright et al.	Serious	Low	Serious	Low	Low	Low	Low

Low	Moderate	Serious	Critical

(C) Confounding bias
(S) Selection bias
(ME) Measurement of Exposure
(DE) Departures from exposure
(MI) Missing data
(MO) Measurement of Outcomes
(R) Reported results

Table S4. Outcome data for studies assessing nutrition intake, nutrition adequacy, nutrient content of the meals, and meal consumption.

Outcomes	Study	**Nutrition Intake**				**Dietary Adequacy**		**Nutrition content**		
		**Intervention TMDs**	**Control TMDs**	**Std**	**P value**	**Intervention/ TMDs**	**Control/Std**	**Interventi on/TMDs**	**Control TMDs/S td**	***P* value**
Meal consumption %	Cassen et al.	15% increase								
	de Sa et al. (meal)		B: 75.3<br>S: 74.2	79.7						
	(meal + ONS)		B: 78.0<br>S: 68.9	74.2						
	ONS (morning vs afternoon tea)		B: 82.6 vs 100<br>S: 84.7 vs 96.8	81.9 vs 58.3						
	Farrer et al.	↑ %participants consuming from < 25% to >75% in 2-week of moulded puree ***p* = 0.03**<br>NS ↓ in plate wastage with moulded compared to non-moulded puree (160g vs 286g, *p* = 0.09)								
	Higashiguchi	Enzyme-infused TMDs showed a slightly ↑ consumption compared to unmodified TMDs (69.6% vs 68.7%, *p* > 0.05)								
	Keller et al.	NS ↑ by using mix of cMTF and rMTF compared to cMTF (*p* = 0.1)								
	Kennewell and Kokkinakos	NS ↑ with infant cereal fortified puree								
	Miles et al. `		P: 59%<br>MM: 55%<br>S: 52%	43%						
	Torrence	Significant ↑ consumption of breakfast (p=.007), dinner (p=.017) and dessert (p=.005) with pre-shaped puree								
	Wright et al. `		13% (n=4)							

	Zanini et al.		83.4% and 12.3% of the texture-individualised TMDs were fully or partially consumed respectively<br>4.1% of the meals were not eaten								
% of Energy eaten	Nowson et al.			S: 76.7 (24.5)<br>P: 74.1 (19.8)	82.2 (16.9)						
Energy (kcal/d)	Bannerman and McDermott			1312 (326)	1569 (260)	**0.02**	Both were sig. less than DRI, $p < 0.001$				
	Beck and Hansen``						Chopped diet menu below DRI of 2100 kcal/d, NS		C: 2050<br>B: 2170	2400	
	Cassen et al.		41% increased intake								
	Dahl et al.			1074 (202)							
	Durant								1666 (78)	2120 (222)	**< 0.001**
	Foley et al. kcal/kg/d	Day 1		19.2 (5.1)	20.0 (5.4)	NS	79.6 (18.6) %	81.9 (20.0) %			
		Day 7		18.1 (6.2)	19.4 (5.9)	NS	75.7 (24.1) %	77.0 (17.8) %			
		Day 11		20.6 (6.0)	20.5 (5.0)	NS	86.1 (27.0) %	79.0 (20.9) %			
		Day 14		22.0 (5.0)	21.3 (6.2)	NS	94.0 (18.0) %	81.3 (18.2) %			
		Day 21		19.4 (6.2)	22.3 (9.0)	NS	84.1 (23.4) %	84.1 (28.6) %			
	Germain et al.		1947 (317)	1603 (366)		0.08	Both pre-/post intervention had **inadequate** intake (2000 kcal/d)				

healthcare MDPI

	Higashiguchi	1097.2 (395)	1036.4 (349.3)		**<0.05**	Both pre-/post intervention had **inadequate** intake (1200-1500 kcal/d)	1365		
	Johnson et al.		1291 (140)	1380 (207)	0.100	Both diets had **inadequate** intake (1900 kcal/d)	1786	2153	
	Keller et al. (per plate)	142.7 (35.3)	143.1 (35.3)		1.0				
	Massoulard et al.		C: 1764.3 (283.2) M: 1499.2 (308.8)	1627.0 (447.1)		All diets had **inadequate** intake (30-40 kcal/kg/d)			
	McCormick et al. ``		582 (241)	267 (50)	**0.0001**				
	Nowson et al.		S: 932.1 (501.9) P: 908.2 (167.3)	1123.3 (454.1)	**<0.001**				
	Ott et al.	1611.1	1417.7		**<0.05**	Adequate (1630 kcal/d) intake after shaped and fortified. Inadequate intake pre-intervention.			
	Philip et al.	1534 (310)	1305 (282)			Both groups had appropriate intake (1029-1326 kcal/d)	2462 (310)	2057 (294)	
	Reyes-Torres et al. (kcal/kg/d)	40 (15)	34 (10)		0.11				
	Sherwin et al.		S: 908.2 (47.8) H: 1027.7 (47.8)	1338.4 (47.8)	**<0.05**				

healthcare

	Taylor and Barr	1342 (177)	1325 (207)		0.565				1651 (177)	1661 (185)	NS
	Vucea et al.								1800.9 (507.2)	2058.4 (397.1)	NS
	Welch et al.	2460.5 (93.1)	1662.5 (121.6)		**<0.001**	Adequate (1900kcal/d) with fortified cereal and ONS<br>Inadequate intake pre-intervention					
	Wright et al.<br>% of requirements meet		927 (339)	1462 (615)	**<0.0001**	-609 (255)<br>60%	-85 (565)<br>95%	**<.0001**			
	Zanini								1850		
Protein (g/d)	Bannerman and McDermott		44.4 (12.4)	49.6 (10.4)	0.23	Both met the DRV					
	Beck and Hansen ``<br>% of energy content					All below DRV of 18% E (90g), **p<.001**			C: 12<br>B: 11	13%	
	Cassen et al.	36% increased intake									
	Dahl et al.		54 (19)			43% and 87% of pureed diets from 2 provinces were able to provide 59g/d (moderate risk of inadequacy); 0% and 40% were able to provide 78g/d (low risk of inadequacy)			57.9 (7.9)<br>85.4 (31.1)		
	Durant								67 (2)	74 (7)	**<.001**

	Foley et al. g/kg/d	Day 1		0.69 (0.17)	0.84 (0.28)	NS	Both diets had inadequate intake (1.0 g/kg/d)			
		Day 7		0.67 (0.28)	0.85 (0.27)	NS				
		Day 11		0.76 (0.26)	0.87 (0.22)	NS				
		Day 14		0.89 (0.19)	0.80 (0.23)	NS				
		Day 21		0.71 (0.29)	0.90 (0.31)	NS				
	Germain et al.		83.1 (21.2)	56.6 (19.8)		**.03**	Met lower limit (1.0-1.3 g/kg/d) pre-intervention			
	Higashiguchi		49.9 (18.1)	40.0 (14.1)		**<.01**		64.1		
	Johnson et al.			56.0 (7.0)	56.0 (11.0)	0.849	Both diets had adequate intake (46.0 g/d)	78.0	88.0	
	Keller et al. (per plate)		10.6 (5.0)	9.7 (5.0)		0.4				
	Massoulard et al.			C: 69.7 (10.2)<br>M: 68.0 (15.7)	62.4 (16.8)		Lower limit DRI (1.0g/kg/d)			
	McCormick et al. ``			15 (7)	7 (3)	**0.001**				
	Nowson et al.			S: 46.8 (18.8)<br>P: 41.7 (15.2)	48.9 (18.0)	NS				
	Ott et al.		69.0	40.4		**<0.01**	Adequate (60 g/d) intake after shaped and fortified<br>Inadequate intake pre-intervention			
	Philip et al. (g/kg/d)		1.04 (0.29)	1.00 (0.30)			↓ risk of inadequate intake (0.8 g/kg/d) was in fortified TMDs (8% vs 16%)	1.93 (0.37)	1.54 (0.39)	

	Reyes-Torres et al.(g/kg/d)	1.8 (0.7)	1.4 (0.5)		0.35				19 (5)% E	17 (5)%E	
	Sherwin et al.		S: 49.8 (3.1)<br>H: 39.5 (1.9)	62.2 (2.9)	**<0.05**						
	Vucea et al.					Both diets provided adequate DRV (56 g/d)			82.2 (23.6)	86.5 (20.7)	NS
	Welch et al. (g/kg/d)	2.33 (0.09)	1.58 (0.11)		**<0.0001**	Both diets met DRV (0.8g/kg/d)					
	Wright et al.<br>% of requirements meet		40 (18.6)	60 (27)	**0.003**	-22 (16.9) 55%	-6 (24.8) 91%	**.013**			
	Zanini								65.81<br>13.08 % E		
NSP (g/d)	Bannerman and McDermott		6.3 (1.7) ~ 35% DRV	8.3 (2.7) ~ 46% DRV	**0.03**	Both were sig. less than DRI (19g/d), **p<.001**					
Carbohydrates (g/d)	Durant								255 (6)	320 (38)	**<.001**
	Germain et al.	272 (44.5)	254 (66.8)		0.55						
	Higashiguchi	166.7 (61.6)	161.4 (54.9)		>0.05				207		
	Keller et al. (per plate)	14.6 (4.9)	15.8 (4.9)		0.3						
	Massoulard et al. %E		C: 48.0 (5.5)<br>M: 46.7 (5.2)	45.5 (7.2)							
	Reyes-Torres et al. %E								56 (11) %E	55 (12)% E	

	Vucea et al.					Both diets provided adequate DRI (130 g/d)	229.2 (66.9)	265.8 (52.2)	NS
	Zanini						259.04 56.01% E		
Fibre (g/d)	Germain et al.	12.2 (4.01)	16.2 (4.57)		0.1				
	Keller et al. (per plate)	1.8 (0.9)	1.9 (0.9)		0.6				
	Nowson et al.		S: 15.3 (6.5) P: 14.6 (4.9)	19.2 (10.4)	**<0.01** between P & Std.	Insufficient, 73% consumed <20g/d			
	Sherwin et al.		S: 14.0 (1.2) P: 16.5 (0.9)	20.8 (1.5)	**<0.05** between S & Std.				
	Vucea et al.					Both diets provided **inadequate** DRI (30 g/d)	16.9 (5.68)	20.9 (5.05)	NS
	Welch et al.	12.0							
Fat (g/d)	Beck and Hansen `` % of energy content					All below DRI of 50% E, **p<.001**	C: 38 B: 37	40%	
	Durant						43 (7)	63 (9)	**<.001**
	Germain et al.	62.3 (11.2)	43.3 (11.1)		**<0.01**				
	Higashiguchi	25.2 (9.8)	25.3 (10.8)		>0.05		29.7		
	Keller et al. (per plate)	5.5 (1.3)	4.6 (1.3)		**0.0**				
	Massoulard et al. %E		C: 33.8 (6.1) M: 34.3 (5.4)	37.8 (6.9)	**<0.05**				
	Reyes-Torres et al. %E						26 (9) %E	28 (9) %E	

	Zanini							63.55<br>30.91 %E		
Fluid (ml/d)	Bannerman and McDermott		1196 (288) ~ 75% estimated requirement	1611 (362)	**0.002**	TMDs were sig. **less** than EAR, **p<.001**; 33.3% (n=5) on Std, and 6.7% (n=1) on TMD met requirements				
	Philip et al.	301 (103)	301 (103)			100% risk of **inadequate** intake (1500ml/d) in both groups			594 (209)	
	Taylor and Barr	698 (156)	612 (176)		**0.003**			1148 (177)	1116 (387)	NS
			**Dysphagia diet + TF**	**Enteral feed → Dysphagia diet + TF**		**Dysphagia diet+TF**	**Enteral feed → Dysphagia diet + TF**			
	Finestone et al.		755 (162)	984 (486)		Met 33 (5) % of fluid requirements (2278 (331) ml/d)	Met 43 (20) % of fluid requirements (2294 (276) ml/d)			
		**TF+ water**	**TF only**							
	Garon et al.	1318 (855 + 463)	1210	p = **0.03 on TF,** NS on overall intake						

	Karagiannis et al.	Intervention vs control	1767 (10.7)	1378 (33.7)		**<0.001**						
		post- vs pre-	1767 (10.7)	1428 (7.0)		**≤0.001**						
				**TMD +TF +External fluid**	**TMD +TF**							
	Vivanti et al.			2165 (867)	1174 (455)	**<0.001**	**Inadequate** by TMD+TF only					
			**Pre-thickened**	**Powder-thickened**								
	McCormick et al. ``		795 (346)	785 (202)		0.47						
	Whelan et al.		554 (173)	278 (233)		**0.04**	Mean daily intake was 455 ml/d, only meet 22% of the requirements					
			547 (391)	490 (484)		NS						
Na (mg)	de Sa et al. % of inadequacy * (>UL)						S: 80.0<br>B: 85.2	76.2	**.009**			
	Germain et al.		3270 (915)	2781 (297)		0.32						
	Higashiguchi		2302.4 (873.9)	2261.8 (989.5)		>0.05						
	Keller et al. (per plate)		323.8 (114.5)	257.2 (114.5)		0.2				2042		
	Vucea et al.						Both diets provided adequate DRI (2300 mg/d)			2775.9 (920.3)	3140.7 (830.2)	NS
K (mg/d)	de Sa et al. % of inadequacy						S: 100.0<br>B: 100.0	100.0	**N/A**			

	Germain et al.	3913 (665)	309 (689)		**0.04**						
	Johnson et al.		2148 (322)	2116 (492)	0.798	Both diets met the DRI (2000mg/d)			2988	3064	
	Vucea et al.					Both diets provided **inadequate** DRI (4700 mg/d)			3111.3 (1035.6)	3103.2 (768.3)	NS
Fe (mg/d)	de Sa et al. % of inadequacy					S: 0.0 B: 40.7	19.8	**0.027**			
	Germain et al.	15.6 (4.34)	13.9 (3.95)		0.45						
	Johnson et al.		8.0 (1.0)	10.0 (3.0)	**0.002**	TMD had **inadequate** intake (10.0 mg/d)			10.3	14.8	
	Kennewell and Kikkinakos (per meal)					Increased 57% iron content			P:4.22 MM: 4.11	2.69 2.62	
	Moreira et al. **					All diets contained **insufficient** DRI (18 mg/d)			S:16.9 B:9.3	13.0	**<.05**
	Philip et al.	34.0 (7.0)	16.0 (3.0)			Appropriate intake (10 mg/d) in both groups			54 (11)	20 (4)	
	Vucea et al.					Both diets provided adequate DRI (9 mg/d)			11.3 (3.17)	13.6 (3.32)	**<.01**
	Welch et al.	22.9 (1.14)	18.1 (1.29)		**<0.0007**	Adequate (10 mg/d)	Adequate				
Zn (mg/d)	de Sa et al. % of inadequacy					S: 20.0 B: 48.2	69.0	**<.001**			
	Germain et al.	14.6 (4.42)	7.69 (3.44)		**<0.01**						

healthcare MDPI

	Johnson et al.		6.1 (1.3)	6.8 (2.0)	0.174	Both diets did not meet the DRI (12.0mg/d)	8.6	9.7	
	Moreira et al. **					Both standard and blend contained insufficient Zn content (11 mg/d)	S:21.0 B:10.48	8.5	**<.05**
	Vucea et al.					Both diets provided **inadequate** DRI (11 mg/d)	9.31 (3.88)	10.6 (3.22)	**<.01**
	Welch et al.	18.1 (0.7)	10.0 (0.6)		**<0.0001**	Adequate (12 mg/d)<br>Inadequate			
Ca (mg/d)	de Sa et al. % of inadequacy					S: 40.0 B: 88.9<br>76.2<br>**.006**			
	Germain et al.	1347 (644)	865 (257)		0.1				
	Johnson et al.		667 (170)	660 (243)	0.916	Both diets did not meet the DRI (800mg/d)	1241	1342	
	McCormick et al. ``		544 (156)	25 (8)	**0.0001**				
	Nowson et al.		S: 366.1 (180.6) P: 356.8 (155.8)	437.9 (127.2)	NS	All diets had inadequate intake, 94% consumed <75% RDI			
	Philip et al.	1310 (332)	507 (120)			↓ prevalence of inadequate intake (800 mg/d) in fortified TMDs (9 vs 95%)			
	Sherwin et al.		S: 405 (50) H:513 (41)	544 (27)	NS				
	Vucea et al.					Both diets provided **inadequate** DRI (1200 mg/d)	1031.4 (459.4)	1016.3 (373.8)	NS

	Welch et al.	2272 (51.2)	888.8 (71.2)		**<0.001**	Both diets met DRV (800mg/d))					
Mg (mg/d)	de Sa et al. % of inadequacy					S: 90.0<br>B: 85.2	59.5	.064			
	Germain et al.	366 (92.2)	253 (74.1)		**0.02**						
	Vucea et al.					Both diets provided **inadequate** DRI (420 mg/d)			265.4 (94.7)	315.1 (74.6)	**<.01**
	Welch et al.	521.3 (12.9)	208.8 (13.2)		**<0.0001**	Adequate (280/350 mg/d)	Inadequate				
Cu (mg/d)	de Sa et al. % of inadequacy					S: 70.0<br>B: 40.7	42.1	0.563			
	Moreira et al. **					Soft diet contained insufficient Cu content (0.9 mg/d)			S:0.71<br>B: 1.00	0.97	**<.05**
	Vucea et al.					Both diets provided adequate DRI (0.9 mg/d)			1.08 (0.55)	1.41 (0.88)	NS
Mn (mg/d)	de Sa et al. % of inadequacy					S: 80.0<br>B: 37.0	24.6	**.004**			
	Moreira et al. **					Soft diet contained insufficient Mn content (2.3 mg/d)			S:1.72<br>B: 3.09	3.34	NS
	Vucea et al.					Both diets provided adequate DRI (2.3 mg/d)			2.98 (1.38)	4.32 (1.38)	NS
P (mg/d)	de Sa et al. % of inadequacy					S: 10.0<br>B: 11.1	17.5	0.409			

	Vucea et al.					Both diets provided adequate DRI (700 mg/d)	1355.8 (440.1)	1465.9 (362.3)	NS
	Welch et al.	2520 (64.8)	1307.2 (84.8)		**<0.0001**	Both diets met DRV (800 mg/d)			
Se (ug/d)	de Sa et al. % of inadequacy					S: 10.0 B: 33.3	22.2	0.524			
	Moreira et al. **					Blend diet provided inadequate DRI (55 ug/d)	S:0.06 B: 0.05	0.07	**NS**
	Vucea et al.					Both diets provided adequate DRI (55 ug/d)	0.874 (0.445)	1.163 (0.352)	NS
Vitamin A (ugRE/d)	Philip et al.	1705 (31)				Appropriate intake (800/1000 RAE/d) in both groups			
	Vucea et al.					Both diets provided adequate DRI (900 RAE /d)	982.4 (503.9)	1061.8 (618.0)	NS
	Welch et al.	2133.9 (72.9)	891.9 (72.9)		**<0.0001**	Both diets met DRV (800/1000 RAE/d)			
Vitamin C (mg/d)	Adolphe et al.	228 (67)	151 (78)		**0.007**	All met DRI (90 mg/d)	90% met DRI			
	Germain et al.	175.0 (44.4)	182.0 ()76.1		**0.82**				
	Johnson et al.		104.0 (18.0)	89.0 (29.0)	**0.027**	Both diets met the DRI (60 mg/d)	113.0	124.0	
	McCormick et al. ``		170 (134)	35 (48)	**0.001**				
	Philip et al.	117 (39)				Appropriate intake (60mg/d) in both groups	184 (38)	184 (38)	
	Vucea et al.					Both diets provided adequate DRI (90 mg/d)	128.6 (69.1)	130.6 (74.0)	NS

	Welch et al.	180.2 (5.4)	78.6 (6.42)		**<0.0001**	Both diets met DRV (60mg/d)				
Vitamin D (ug/d)	Adolphe et al.	12.2 (3.3)	2.1 (2.2)		**0.005**	0% met DRI pre-/post-fortification (15 ug/d)				
	Germain et al.	10.1 (5.35)	5.19 (2.01)		**0.05**					
	Johnson et al. (IU/d)		131 (51)	157 (77)	0.209	Both diets did not meet the DRI (200 IU/d)		396	445	
	McCormick et al. ``		5 (2)	0 (0)	**0.00001**					
	Nowson et al.		S: 0.8 (0.5) P: 1.9 (0.5)	1.0 (0.8)	NS	Insufficient, 91% consumed <3 ug/d				
	Vucea et al.					Both diets provided **inadequate** DRI (20 ug/d)		8.42 (4.43)	7.52 (3.78)	**<0.01**
	Welch et al. (IU/d)	614 (14)	175 (20.2)		<0.0001	Adequate (200IU/d)	Inadequate			
Vitamin E (mg/d)	Johnson et al.		12.0 (2.4)	13.0 (3.8)	0.256	Both diets met the DRI (8.0 mg/d)		16.0	21.0	
	Vucea et al.					Both diets provided **inadequate** DRI (15 mg/d)		5.46 (2.68)	6.74 (2.40)	**<0.01**
	Welch et al.	21.72 (0.65)	4.5 (0.46)		**<0.0001**	Adequate (8.0 mg/d)	Inadequate			
Thiamin (mg/d)	Adolphe et al.	1.7 (0.3)	0.8 (0.3)		**0.005**	All met DRI (1.2 mg/d)	10% met DRI			
	Germain et al.	1.92 (0.68)	1.54 (0.4)		0.2					
	Johnson et al.		1.0 (0.2)	1.3 (0.3)	**0.007**	Both diets met the DRI (1.0 mg/d)		1.4	1.8	

	Philip et al.	2.33 (0.63)	0.80 (0.20)			↓ prevalence of inadequate intake (1.0/1.2 mg/d) in fortified TMDs (0 vs 57%)		3.91 (1.10)	1.18 (0.38)	
	Vucea et al.					Both diets provided adequate DRI (1.2 mg/d)		1.38 (0.60)	1.67 (0.50)	NS
	Welch et al.	2.56 (0.08)	1.24 (0.08)		**<0.0001**	Both diets met DRV (1.0 mg/d)				
Riboflavin (mg/d)	Adolphe et al.	2.2 (0.4)	1.2 (0.4)		**0.005**	All met DRI (1.3 mg/d)	30% met DRI			
	Germain et al.	3.00 (1.22)	1.78 (0.56)		**0.02**					
	Johnson et al.		1.4 (0.2)	1.5 (0.4)	0.083	Both diets met the DRI (1.2mg/d)		2.2	2.6	
	Philip et al.	2.98 (0.74)	1.2 (0.3)			↓ prevalence of inadequate intake (1.2/1.4 mg/d) in fortified TMDs (0 vs 28%)		5.00 (1.11)	1.83 (0.44)	
	Vucea et al.					Both diets provided adequate DRI (1.3 mg/d)		2.28 (0.94)	2.43 (1.03)	NS
	Welch et al.	3.96 (0.11)	1.82 (0.12)		**<0.0001**	Both diets met DRV (1.2 mg/d)				
Niacin (mg/d)	Adolphe et al.	24.0 (6.0)	19.0 (5.0)		**0.007**	All met DRI (16 mg/d)	70% met DRI			
	Germain et al.	36.2 (10.9)	22.2 (8.01)		**0.01**					
	Philip et al.	27.35 (6.81)	13.5 (3.4)			↓ prevalence of inadequate intake (13/15 mg/d) in fortified TMDs (0 vs 55%)		43.67 (9.78)	19.03 (4.52)	
	Vucea et al.					Both diets provided adequate DRI (16 mg/d)		28.8(12.3)	34.8 (9.85)	NS

healthcare

MDPI

	Welch et al.	29.89 (1.08)	21.45 (1.69)		**<0.0001**	Both diets met DRV (13/15 mg/d)				
Vitamin B6 (mg/d)	Adolphe et al.	2.7 (0.6)	1.6 (0.6)		**0.007**	All met DRI (1.7 mg/d)	30% met DRI			
	Johnson et al.		1.1 (0.3)	1.4 (0.5)	**0.029**	Both diets did not meet the DRI (1.6 mg/d)		1.2	1.6	
	Vucea et al.					Both diets provided **inadequate** DRI (1.7 mg/d)		1.48 (0.62)	1.68 (0.52)	NS
	Welch et al.	1.70 (0.04)	1.34 (0.10)		**<0.006**	Adequate (1.6 mg/d)	Inadequate			
Folacin (ug/d)	Adolphe et al.	505 (86)	114 (58)		**0.005**	All met DRI (400 ug/d)	0% met DRI			
	Johnson et al.		166 (22)	189 (62)	0.069	TMD had **inadequate** intake (180 ug/d)		214	281	
	Philip et al.	160 (52)				47 (female)/97% (male) risk of **inadequate** intake (180/200 ug/d) in both groups		264 (71)	264 (71)	
	Vucea et al.					Both diets provided **inadequate** DRI (400 ug/d)		267.8 (117.5)	375.0 (126.1)	**<0.01**
Vitamin B12 (mg/d)	Adolphe et al.	5.1 (1.4)	3.4 (1.2)		**0.007**	All met DRI (2.4 mg/d)	90% met DRI			
	Johnson et al.		3.2 (0.8)	3.6 (1.3)	0.219	Both diets had adequate intake (2.0 mg/d)		5.1	6.2	
	Vucea et al.					Both diets provided adequate DRI (2.4 mcg/d)		5.13 (3.04)	5.55 (4.89)	NS

healthcare

	Welch et al.	6.41 (0.18)	1.78 (0.17)		**<0.0001**	Adequate (2.0 mg/d)	Inadequate			
Pantothenic Acid (mg)	Adolphe et al.	5.7 (1.0)	3.1 (1.1)		**0.005**	90% met DRI (5 mg/d)	0% met DRI			
	Vucea et al.					Both diets provided adequate DRI (5 mg/d)		10.4 (33.0)	22.3 (49.5)	**<0.01**
Vitamin K (mcg)	Vucea et al.					Both diets provided **inadequate** DRI (120 mcg/d)		88.2 (95.3)	1103 (101.1)	**<0.01**

Note. ` Percentage of participants completed 100% of the meal

`` Results from Beck and Hansen McCormick et al. study was expressed as Median (Interquartile Range)

* % of inadequate intake including either lower than Estimated Average Requirements or above Upper Limits

** Results from Moreira et al. study was the mean value calculated from 3 occasions

TMD – Texture modified diet; TF – Thickened fluid; S - Soft diet; B – Blend diet; P- Pureed diet; C – Chopped diet; M – Mixed diet; MM – Minced and moist; H - Homogenised; Std – Standard diet; ONS – Oral nutrition supplement; DRI – Dietary reference intake

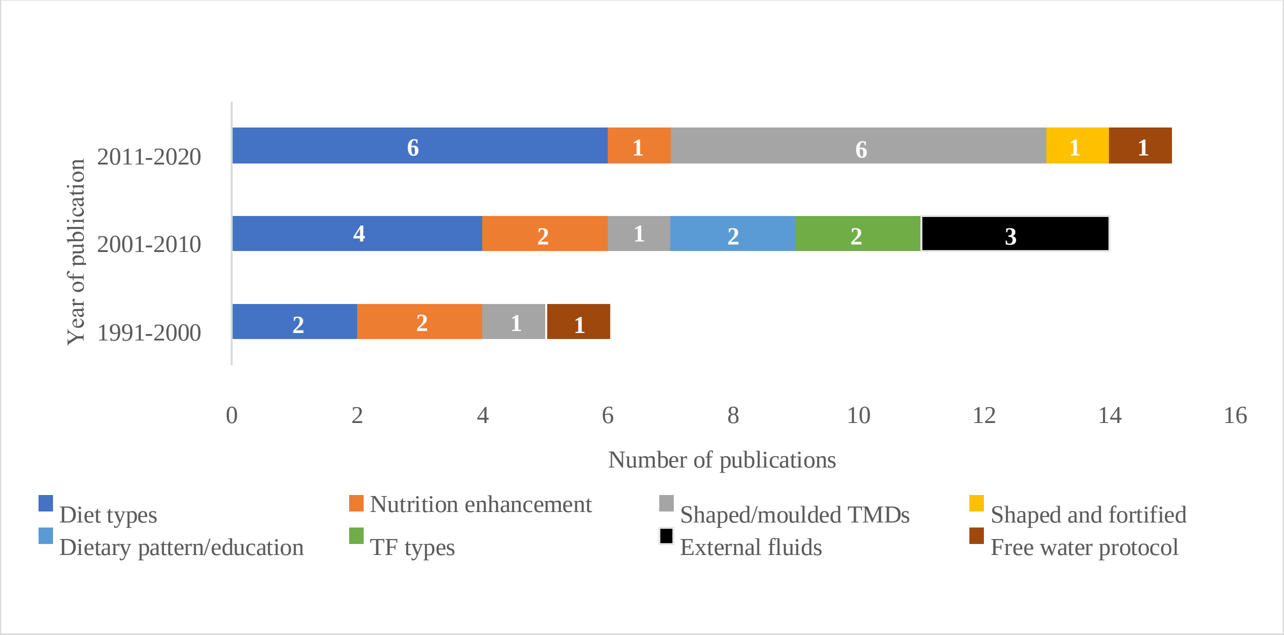

Figure S1. Eligible studies timeline.

Note. Nutrition enhancement includes oral nutrition supplements and nutrient fortification; TMDs–Texture 4 modified diets; TF–Thickened fluids; External fluids includes enteral feeds and intravenous therapy; Free water 5 protocol regarding free access of water for patients on TFs.

	Regular diets			Texture modified diets				Mean Difference
Study or Subgroup	Mean	SD	Total	Mean	SD	Total	Weight	IV, Random, 95% CI
Bannerman and McDermott	1,596	260	15	1,312	326	15	8.4%	284.00 [72.98, 495.02]
Johnson et al.	1,380	207	31	1,291	140	20	13.2%	89.00 [-6.26, 184.26]
Massoulard et al.	1,627	447.1	49	1,764.3	283.2	12	8.6%	-137.30 [-340.64, 66.04]
Massoulard et al.	1,627	477.1	49	1,499.2	308.8	26	9.6%	127.80 [-50.90, 306.50]
Nowson et al.	1,123.3	454.1	114	932.1	501.9	48	10.2%	191.20 [26.55, 355.85]
Nowson et al.	1,123.3	454.1	114	908.2	167.3	53	13.2%	215.10 [120.35, 309.85]
Sherwin et al.	1,338.4	47.8	36	908.2	47.8	13	15.2%	430.20 [399.89, 460.51]
Sherwin et al.	1,338.4	47.9	36	1,027.7	47.8	26	15.3%	310.70 [286.57, 334.83]
Wright et al.	1,462	615	25	927	339	30	6.5%	535.00 [265.12, 804.88]
**Total (95% CI)**			**469**			**243**	**100.0%**	**230.90 [140.79, 321.02]**

Heterogeneity: $Tau^2 = 13705.01$; $Chi^2 = 104.70$, $df = 8$ ($P < 0.00001$); $I^2 = 92\%$
Test for overall effect: $Z = 5.02$ ($P < 0.00001$)

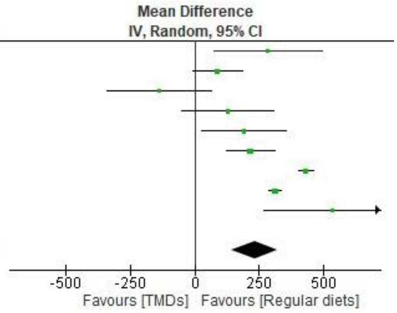

Figure S2. Forest plot of the effect of texture modified diets and regular diets on energy intake (kJ/d) among older adults.

	Regular diets			Texture modified diets				Mean Difference
Study or Subgroup	Mean	SD	Total	Mean	SD	Total	Weight	IV, Fixed, 95% CI
Johnson et al.	660	243	31	667	170	20	1.5%	-7.00 [-120.44, 106.44]
Nowson et al.	437.9	127.2	114	356.8	155.8	53	8.4%	81.10 [33.09, 129.11]
Nowson et al.	437.9	12.72	114	366.1	180.6	48	7.4%	71.80 [20.66, 122.94]
Sherwin et al.	544	27	36	513	41	26	59.1%	31.00 [12.94, 49.06]
Sherwin et al.	544	27	36	405	50	13	23.6%	139.00 [110.42, 167.58]
**Total (95% CI)**			**331**			**160**	**100.0%**	**63.14 [49.26, 77.03]**

Heterogeneity: $Chi^2 = 41.36$, $df = 4$ ($P < 0.00001$); $I^2 = 90\%$
Test for overall effect: $Z = 8.91$ ($P < 0.00001$)

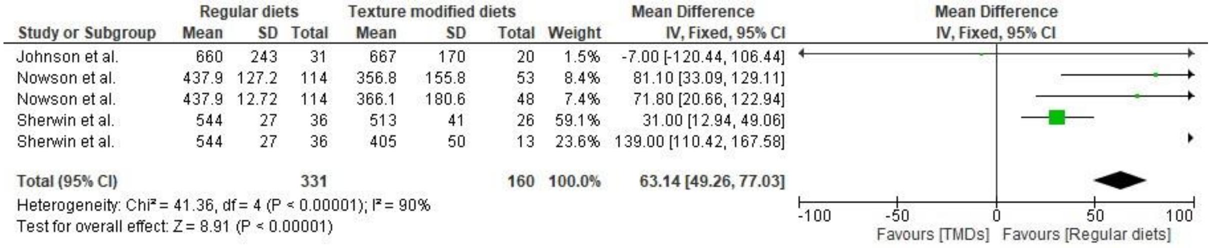

Figure S3. Forest plot of the effect of texture modified diets and regular diets on calcium intake (mg/d) among older adults.

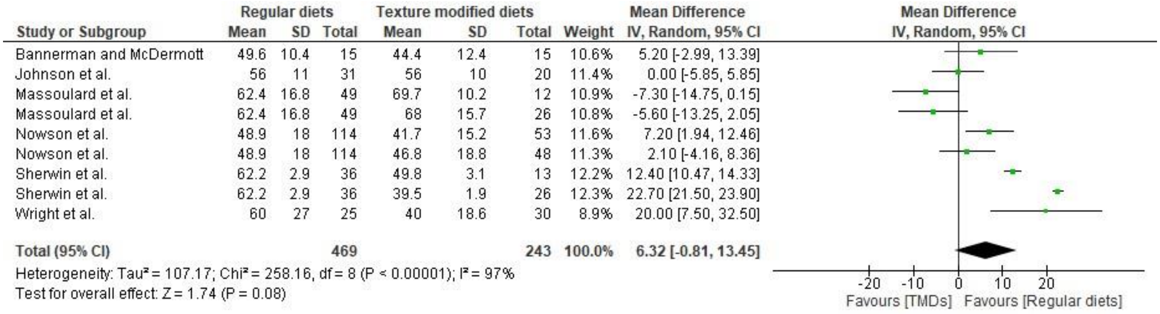

Figure S4. Forest plot of the effect of texture modified diets and regular diets on protein intake (g/d) among older adults.

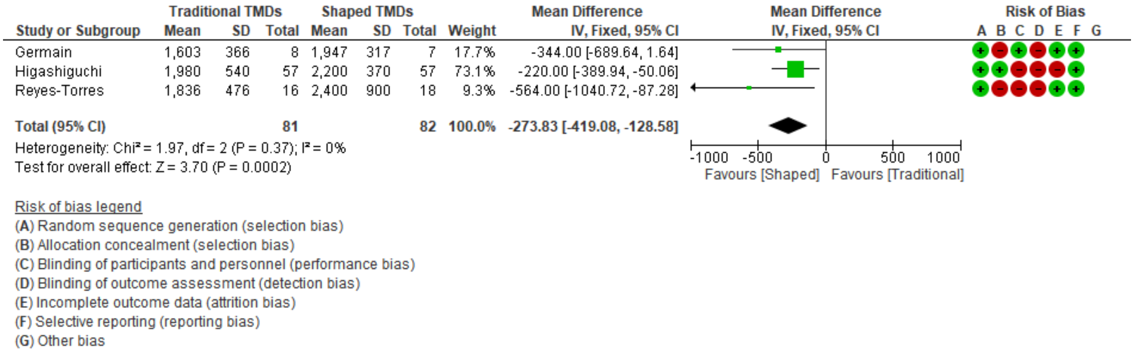

Figure S5. Forest plot of the effect of shaped and traditional texture modified diets on energy intake (kcal/d) among older adults.

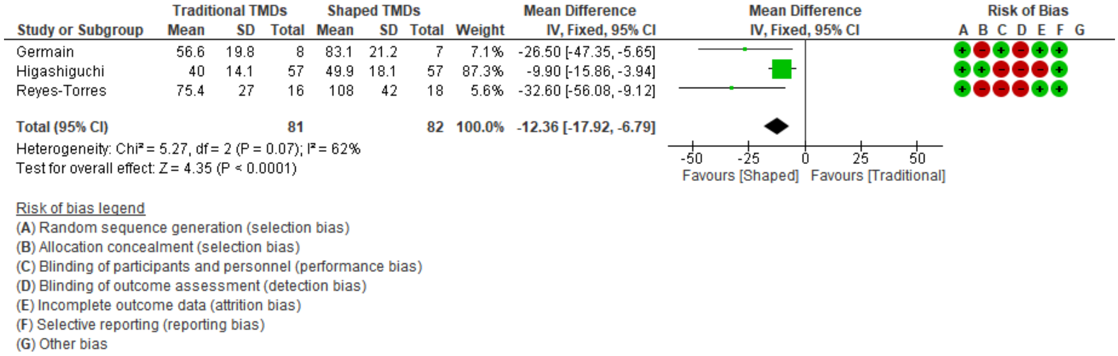

Figure S6. Forest Plot of the effect of shaped and traditional texture modified diets on protein intake (g/d) among older adult.
